# Safety and pharmacokinetic profile of apixaban in end‐stage renal disease: A real‐world analysis

**DOI:** 10.1002/jha2.606

**Published:** 2022-11-29

**Authors:** Chen I. Tseng, Alistair J. Roddick, Matthew J. Bottomley, Susan Shapiro, Udaya Udayaraj

**Affiliations:** ^1^ Oxford Kidney Unit, Churchill Hospital Oxford University Hospitals NHS Foundation Trust Oxford UK; ^2^ Oxford Haemophilia and Thrombosis Centre, Churchill Hospital Oxford University Hospitals NHS Foundation Trust Oxford UK; ^3^ Nuffield Department of Medicine University of Oxford Oxford UK; ^4^ Radcliffe Department of Medicine University of Oxford Oxford UK; ^5^ Oxford NIHR Biomedical Research Centre Oxford UK

**Keywords:** anticoagulation, apixaban, atrial fibrillation, dialysis, end stage renal disease, pharmacokinetics

1

Vitamin K antagonists (VKA) remain the primary treatment of non‐valvular atrial fibrillation (NVAF) in patients with end‐stage renal disease on dialysis for stroke prevention. However, the evidence of benefits for using VKA in dialysis patients remains inconclusive [1]. VKAs pose problems in this cohort due to risk of calciphylaxis and labile international normalised ration (INR), which may lead to adverse outcomes [2, 3]. Sub‐optimal anticoagulation control on VKA strongly correlates with increased risks of thromboembolic and bleeding events [4].

Apixaban clearance is less dependent on renal function compared to other direct‐acting oral anticoagulants. Data from retrospective studies suggest that apixaban has at least similar efficacy in stroke prevention for NVAF, but lower bleeding risk compared to VKA in dialysis cohorts [5, 6]. Nevertheless, apixaban is currently only licensed for use in dialysis patients in the US but not in the UK or Europe.

This was a single‐centre prospective analysis carried out at Oxford Kidney Unit to evaluate the efficacy and safety of apixaban when used according to the local guideline at the dose of 2.5‐mg twice daily (BD) for dialysis patients with NVAF (Supporting Information). As apixaban is not licensed for use in dialysis patients in the UK, the off‐licence use was discussed with patients by supervising nephrologist prior to treatment initiation.

All patients established on either peritoneal (PD) or haemodialysis (HD) who were commenced on apixaban between January 2020 and December 2021 were included. Clinical outcomes data were collected to December 2021. Electronic patient records were reviewed for clinical outcomes of thromboembolism, stroke, haemorrhage or death. Bleeding episodes were assessed for severity with reference to the International Society on Thrombosis and Haemostasis bleeding scale [7]. Events were not formally adjudicated. Causes of mortality were reviewed for potential relation to apixaban.

Apixaban levels in the blood were measured using an automated chromogenic anti‐Xa assay on the Sysmex platform (Sysmex, Milton Keynes, UK). Peak (4 hours post‐dose) and trough apixaban levels were measured at 1 week, 1 month and 3 months after initiating treatment (Supporting Information).

Fifteen patients were included in this analysis. The median (interquartile range [IQR]) duration of follow‐up was 3 (2.5–10.5) months. During the follow‐up period, six (40%) patients died, all for reasons considered unrelated to apixaban (calciphylaxis [*n* = 1], malignancy [*n* = 1], frailty leading to withdrawal of dialysis [*n* = 1], diabetic foot infection [*n* = 1] and ischaemic heart disease [*n* = 2]). Three (20%) patients discontinued apixaban due to change in clinical status: one patient was listed for transplant, whilst two patients reverted to sinus rhythm. Median (IQR) age at commencement was 76 (70–79) years, 11 (79%) were male, 13 (87%) were receiving HD.

Eight (53%) patients completed 3 months of monitoring, of whom two patients did not receive planned 1‐week monitoring due to blood sampling errors. Therefore, six (40%) patients followed the monitoring schedule and remained on apixaban for the duration of follow‐up. Apixaban levels were available for 11 (73%) patients at 1 week, 12 (80%) at 1 month and eight (53%) at 3 months following commencement.

The mean ± standard deviation (SD) for peak and trough levels were 95.7 ± 34.8 ng/ml and 66 ± 34.6 ng/ml, respectively. Seventy‐five percent of recorded peak and trough levels were within the 5th–95th percentile of the reference levels for the general population, who were taking the reduced 2.5‐mg BD dose for NVAF (Figure 1) [8]. Note that 12.5% of measured peak levels and 12.5% of trough levels fell below the respective 5th percentile of the reference range of the general population. No measurements exceeded the 95th percentile. Mean ± SD of peak and trough levels for the two PD patients were 152.2 ± 65.6 ng/ml and 105.3 ± 61.2 ng/ml, respectively. In the case of two patients, trough levels exceeded peak levels.

**FIGURE 1:**
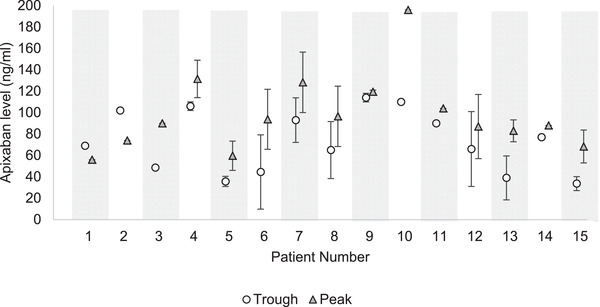
Peak and trough apixaban levels recorded for each patient. Data presented as mean ± standard deviation. Patients without error bars had only one measurement of peak and trough apixaban levels.

In this analysis, most patients peak and trough apixaban levels fell within the respective 5th–95th percentile of the reference range for the general population. This was true irrespective of duration of apixaban treatment, and there was no evidence of supratherapeutic apixaban accumulation over time in six patients who completed three months of monitoring, which is consistent with existing literature (Figure 2) [9]. No cases of stroke or thromboembolic events were observed during follow‐up. Two minor bleeding events were recorded: one patient experienced self‐limiting rectal bleeding, and the other patient noticed blood clots in colostomy bag. Neither patient required intervention, hospitalisation or discontinuation of apixaban.

**FIGURE 2:**
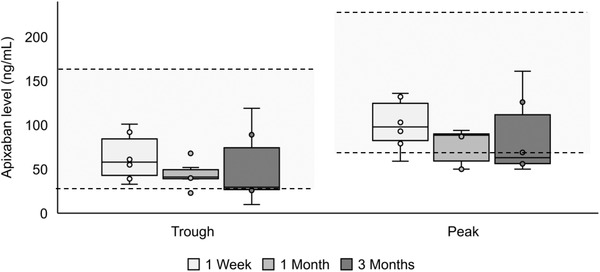
Peak and trough apixaban levels at 1 week, 1 month and 3 months of apixaban therapy. Data presented as box‐and‐whisker plots. Circles denote individual data points.

Pharmacokinetic profiling in this analysis demonstrates there was no cumulative exposure of apixaban in dialysis patients; however 25% of the measurements fell below the 5th percentile; this may be due to dialysis reducing the area under the curve of apixaban. Although there is no known correlation of apixaban levels with efficacy, this does raise the concern that a proportion of patients may risk inadequate dosing of apixaban, in particular when considered alongside a large retrospective study of dialysis patients with NVAF in the US [6]. This found that both 5‐mg BD and 2.5‐mg BD apixaban had reduced the risk of major bleeding compared to warfarin (hazard ratio (HR) 0.71, *p* < 0.03) [6]. Although apixaban 2.5‐mg BD had no difference for stroke risk compared to warfarin, apixaban 5‐mg BD was associated with a lower risk of stroke (HR 0.64, *p* = 0.003) and death (HR 0.64, *p* = 0.003) compared to warfarin [6].

There are no published randomised controlled trials (RCTs) for use of apixaban in dialysis. Results from several ongoing unreported trials may provide definitive evidence on efficacy and safety of apixaban and the optimal dosing strategy in dialysis patients. Of note, a recent RCT demonstrated lower hazard for cardiovascular events and major bleeding with 10‐mg once daily rivaroxaban, compared to VKA in dialysis patients with NVAF [10].

The clinical outcomes in this analysis must be interpreted with caution given the small cohort size, of whom over half discontinued therapy. There was considerable inter‐ and intra‐patient variability in apixaban levels, and in some cases trough levels exceeded peak levels within the same patient. This could be attributed to inaccurate timing of blood sampling or direct effects of dialysis on drug clearance.

This prospective analysis provides additional data to support the use of apixaban at a dose of 2.5‐mg BD in patients receiving dialysis as an alternative to VKA in NVAF, until results from ongoing clinical trials are available. The longitudinal data on apixaban levels, over a 3‐month period, provide reassurance that there is no risk of cumulative exposure to high apixaban levels in dialysis patients.

## AUTHOR CONTRIBUTIONS

CIT performed the data collection and wrote the first draft of the manuscript. MJB, SS, UU and CIT designed the analysis. UU supervised the analysis. AJR and CIT analysed the primary data. AJR, MJB, SS and UU critically reviewed and co‐wrote the manuscript. All authors reviewed and approved the final version of the manuscript

## CONFLICT OF INTEREST

MJB has received educational speaker and conference fees from Astellas and is supported by a Career Development Grant from the Chinese Academy of Medical Sciences (CAMS) Innovation Fund for Medical Science (CIFMS), China (grant number: 2018‐I2M‐2‐002). SS has received educational speaker fees from Bayer and Pfizer, conference support from Bayer and advisory board fees from Pfizer; and funding support from the Medical Research Council (MR/T024054/1). The views expressed are those of the authors and not necessarily those of the NHS, the NIHR or the Department of Health. All the other authors declared no competing interests.

## ETHICS STATEMENT

This manuscript does not contain clinical trial data. Prospective data were collected during routine care and registered as a clinical audit at Oxford University Hospitals NHS Foundation Trust.

## Supporting information



Supporting Information

## Data Availability

The data pertinent to the findings and analysis of this report are available from the corresponding author upon reasonable request.
