# Supplementary material for: Safety and pharmacokinetic profile of apixaban in end‐stage renal disease: A real‐world analysis
Source: EJHaem. 2022 Nov 29;4(1):291–3. doi: 10.1002/jha2.606 (PMC9928643; doi:10.1002/jha2.606)
Supplement: Supplementary file 1 — Supporting Information [file JHA2-4-291-s001.docx]

**SUPPLEMENTARY INFORMATION**

**Supplementary Appendix 1.**

**Supplementary Methods**

This was a single-centre, prospective analysis carried out at Oxford Kidney Unit (OKU) and its satellite dialysis units in network hospitals, part of Oxford University Hospitals NHS Foundation Trust (OUH). As apixaban is not licensed for use in the UK in dialysis patients, a local guideline was developed to allow consideration of apixaban for patients with non-valvular atrial fibrillation (NVAF). This project was undertaken to evaluate this guideline which was approved by the OUH Clinical Effectiveness Committee. VKA remained as the first line agent for anticoagulation in dialysis patients with NVAF, but apixaban could be used (either de novo or as a transition from VKA) in those with: previous VKA-induced skin necrosis or calciphylaxis; where there was more than one risk factor for calciphylaxis; labile INR control whilst on VKA (time in therapeutic range < 65%); or intolerance to VKA.^1^ Apixaban was used at a reduced dose of 2.5mg twice daily (BD) based on existing literature of its use in patients receiving dialysis. Patients were not eligible for apixaban if they were active on the deceased donor renal transplant waiting list, due to the logistical challenge of rapid pre-operative apixaban reversal.

**Supplementary Appendix 2.**

**Apixaban level monitoring**

Monitoring of apixaban activity was performed by assaying apixaban concentration in peripheral blood, which has a linear correlation with the degree of Factor Xa inhibition across a wide range. Apixaban levels were measured using an automated chromogenic anti-Xa assay on the Sysmex platform (Sysmex, Milton Keynes UK). Product specific calibrators allowed reporting across the range 0-600ng/mL. Peak (4 hours post-dose) and trough apixaban levels were measured at 1 week, 1 month and 3 months after initiating treatment. On the day of testing, trough levels were taking prior to commencing dialysis. Peak levels were taken at the end of dialysis. The clearance of apixaban by peritoneal dialysis is unknown, but has been reported to be vary between 4 % and 14% during a haemodialysis session.^2^ Therefore, additional apixaban levels were planned at the point of any change in dialysis modality or haemodialysis session frequency. No dose adjustment of apixaban was planned in the protocol as apixaban levels were being measured primarily to compare results with published data on reference levels in the general population. Measured peak and trough apixaban levels were compared to predicted apixaban steady-state levels when used at a reduced dose of 2.5mg BD for NVAF in the general population, median (5^th^, 95^th^ percentile) 123ng/ml (69, 221) and 79ng/ml (34, 162), respectively.^3^ The dose reduction applies when two of the following three criteria are met: serum creatinine ≥133μmol/L), age ≥80 years or body weight ≤60kg.^3^

**SUPPLEMENTAL REFERENCES**

1. Nigwekar S, Thadhani R, Brandenburg V. Calciphylaxis. New England Journal of Medicine. 2018;378(18):1704-14.

2. Mavrakanas T, Samer C, Nessim S, Frisch G, Lipman M. Apixaban Pharmacokinetics at Steady State in Hemodialysis Patients. Journal of the American Society of Nephrology. 2017;28(7):2241-8.

3. Squibb-Pfizer. B-M. Eliquis 2.5 mg film-coated tablets - Summary of Product Characteristics (SPC): Electronic Medicines Compendium; 2021 [Available from: <https://www.medicines.org.uk/emc/product/4756>].
